# Engineered *Escherichia coli* Modified with Carbon Quantum Dots as a High-Performance Cathode Catalyst for Microbial Fuel Cells

**DOI:** 10.3390/molecules31122039

**Published:** 2026-06-11

**Authors:** Xiangyu Wei, Xiumei Song, Wei Huang, Yating He, Yimin Wang, Pinxiu Liu, Lichao Tan, Lin Yang, Zhongwei Chen

**Affiliations:** 1 Institute of Carbon Neutrality, Zhejiang Wanli University, Ningbo 315100, China; 13298141882@163.com (X.W.); hw15085006041@163.com (W.H.); m15968317286@163.com (Y.H.); 2024881035@zwu.edu.cn (P.L.); tanlcking@163.com (L.T.); 2 Ningbo Key Laboratory of High Energy Density Battery, Yuyao Innovation Institute, Zhejiang Wanli University, Ningbo 315100, China; 18845041835@163.com; 3 Dalian Institute of Chemical Physics, Chinese Academy of Sciences, Dalian 116023, China

**Keywords:** carbon quantum dots, engineered *Escherichia coli*, microbial fuel cells, oxygen reduction reaction, biohybrid catalyst

## Abstract

The strategy of enhancing biocatalytic activity through the modification of natural cells with nanomaterials has overcome the intrinsic catalytic bottlenecks of bacteria, making significant contributions to energy production and pollution treatment. However, chemically engineered biocatalyst systems remain in their early stages of development. Herein, we report a simple and straightforward strategy for constructing an efficient biocatalyst by incorporating carbon quantum dots (CDs) into *Escherichia coli* (*E. coli*) to enhance the oxygen reduction reaction (ORR) at the cathode of microbial fuel cells (MFCs). The introduction of CDs significantly accelerates extracellular electron transfer and metabolic activity, markedly increases intracellular adenosine triphosphate (ATP) levels, and promotes substrate utilization. Furthermore, the engineered *E. coli* exhibits enhanced surface adhesion and increased electronegativity. Electrochemical measurements demonstrate superior ORR activity, delivering a maximum current density of 3.1 mA·cm^−2^ and an onset potential of 0.67 V, outperforming many previously reported biocatalysts. When applied in an MFC system, the modified biocatalyst achieves a maximum power density of 325 μW·cm^−2^, placing it among the highest-performing systems reported to date. This work provides a facile and cost-effective approach for improving MFC performance and offers a promising design strategy for next-generation biohybrid catalysts.

## 1. Introduction

The world is currently confronting critical challenges related to energy shortage and environmental pollution, urgently requiring a transition toward sustainable development models [1]. In this context, harnessing cost-effective and environmentally friendly microorganisms to develop novel green technologies that can simultaneously generate electricity and degrade waste is of both practical and theoretical significance. Microbial fuel cells (MFCs) are a promising example of such a bioelectrochemical system, capable of directly converting the chemical energy stored in waste materials into electricity [2,3,4].

A major challenge in advanced MFC technology lies in the development of efficient, stable, and cost-effective oxygen reduction reaction (ORR) catalysts for the cathode [5]. To address this problem, researchers propose an innovative strategy: engineering microbial cells to function directly as dual-function air cathode catalysts. Unlike conventional noble metal catalysts, microbial catalysts offer distinct advantages, including self-replication and self-repair capabilities, while concurrently degrading organic pollutants. This approach creates a synergistic effect between electricity generation and pollution treatment, highlighting its considerable potential for cost-effective and environmentally sustainable energy applications [6,7,8].

However, the naturally low catalytic activity and limited membrane conductivity of microbial cells restrict their practical application. Therefore, future research should prioritize the interdisciplinary integration of synthetic biology and materials science to rationally engineer microorganisms, such as by constructing “electron transfer highways” to enhance their electrochemical performance [9,10]. Carbon quantum dots (CDs) exhibit high electron mobility, exceptional stability, reliable biocompatibility, and unique optical properties, making them widely applicable in photoluminescence, catalysis, sensing, and biomedicine. Typically synthesized via mild bottom-up hydrothermal approaches using heteroatom-rich precursors such as riboflavin, the resulting CDs feature surfaces abundant in hydroxyl (-OH), carboxyl (-COOH), and amino (-NH_2_) groups, which impart exceptional water solubility and charge-mediating capabilities. Recently, integrating CDs into bioelectrochemical systems, particularly microbial fuel cells (MFCs), has emerged as a compelling strategy to construct high-performance biohybrid catalysts. This integration effectively addresses the intrinsic kinetic bottlenecks of sluggish extracellular electron transfer (EET) and slow cathodic oxygen reduction reaction (ORR). Acting as an “electron highway,” these highly conductive CDs accelerate EET, upregulate intracellular adenosine triphosphate (ATP) levels, and enhance substrate metabolic rates. Furthermore, functionally modified CDs serve as excellent co-catalytic sites to substantially lower the cathodic ORR overpotential, thereby paving the way for cost-effective and sustainable industrial applications of high-performance biocathodes [11,12,13,14,15].

**Figure 1:**
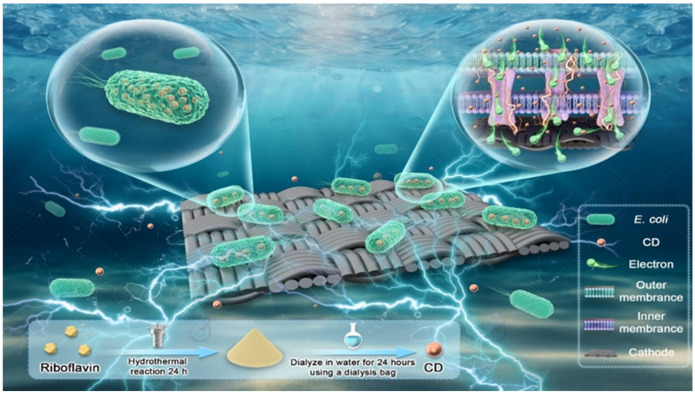
Schematic diagram of carbon dot penetration and interaction within *E. coli* cells.

In this study, water-soluble carbon quantum dots (CDs) were synthesized via a hydrothermal method using riboflavin as a precursor [16,17]. A subsequent simple, green, and efficient dialysis process was employed to select CDs with an appropriate particle size. These CDs were then combined with *Escherichia coli* (*E. coli*), leading to their efficient internalization and the construction of a novel whole-cell biocatalyst (Cell@CDs). The CDs significantly accelerated bacterial metabolism, as evidenced by enhanced intracellular charge, elevated ATP levels, accelerated substrate consumption, heightened extracellular secretory activity, and improved transmembrane and extracellular electron transfer capabilities (Figure 1) [18,19,20]. The introduction of CDs not only significantly enhanced the metabolic activity of *E. coli* but also promoted biofilm formation [21]. Electrochemical tests revealed that this hybrid system exhibited long-term stable power output and generated a markedly higher voltage when applied in MFCs. This study represents the first application of CDs in *E. coli*, providing a simple and effective strategy to enhance electricity generation and electron transfer, thereby demonstrating significant promise for bioenergy and related biocatalytic applications.

## 2. Materials and Methods

### 2.1. Materials

Riboflavin, urea, NaCl, citric acid, Na_2_HPO_4_, KH_2_PO_4_, NH_4_Cl, MgSO_4_, CaCl_2_, D-(+)-glucose, and Nafion™ perfluorinated resin solution were purchased from Aladdin Scientific (Shanghai, China). Tryptone and yeast extract were purchased from OXOID, Thermo Fisher Scientific (Waltham, MA, USA). Acetone, ethanol absolute, sulfuric acid, and nitric acid were purchased from Sinopharm Chemical Reagent (Shanghai, China). SYTO™ 9 Green Fluorescent Nucleic Acid Stains were purchased from Invitrogen™, Thermo Fisher Scientific (Waltham, MA, USA). All chemicals were of analytical grade and used without further purification.

### 2.2. Synthesis of CDs, CDs^EG^, and CDs^U^

The CDs were synthesized via a simple and environmentally friendly hydrothermal method. Briefly, 250 mg of riboflavin was dissolved in 8.75 mL of ultrapure water. The resulting solution was transferred into a 50 mL Teflon-lined stainless-steel autoclave and subjected to a hydrothermal reaction at 180 °C for 12 h. After the reaction, the autoclave was cooled naturally to room temperature (22 ± 2 °C). Then, the resulting crude product was purified by dialysis for 24 h to obtain the final CD sample.

For comparative purposes, two control CD samples were also synthesized:

CDs^EG^: A mixture of 1 g citric acid and 2 g urea in 15 mL ultrapure water was treated hydrothermally at 180 °C for 18 h.

CDs^U^: A total of 1 g of citric acid in 15 mL ultrapure water was treated hydrothermally at 200 °C for 18 h.

### 2.3. Material Characterization

Field emission transmission electron microscopy (TEM, Tecnai F20, FEI, Hillsboro, OR, USA) was employed to obtain high-resolution morphology and element mapping images. The surface morphology of the prepared electrodes was examined using a field emission scanning electron microscope (SEM, Sigma 360, ZEISS, Oberkochen, Baden-Württemberg, Germany). The crystal structure of the composite materials was analyzed by X-ray diffraction (XRD, D8 X-ray diffractometer, BRUKER, Karlsruhe, Baden-Württemberg, Germany). Fourier-transform infrared (FTIR) spectroscopy (Nicolet Is50, Thermo Scientific, Waltham, MA, USA) was used to study the changes in functional groups of the nanofibers before and after carbonization. Structural changes were further identified using Raman scattering spectroscopy (inVia, Renishaw, Gloucestershire, UK) with a 532 nm laser source. Surface elemental composition and oxidation states were determined via X-ray photoelectron spectroscopy (XPS, Thermo Fisher ESCALAB 250Xi, Thermo Fisher Scientific). The viability and spatial distribution of the biofilm were analyzed by laser confocal scanning microscopy (LSM 900, ZEISS).

### 2.4. Construction of the Bioelectrode

Carbon cloth with a geometric area of 1 cm^2^ was sequentially subjected to ultrasonic cleaning in acetone and 99.5% (*v*/*v*) ethanol (15 min each) to remove surface grease and organic impurities. Subsequently, the carbon cloth was dried in an oven at 60 °C for 2 h to completely eliminate residual solvents. The dried carbon cloth was then immersed in a mixed acid solution (concentrated sulfuric acid to concentrated nitric acid at a volume ratio of 3:1) under static conditions for 24 h to enhance its surface hydrophilicity and introduce active sites. Finally, the carbon cloth was thoroughly rinsed with ultrapure water until the wash solution reached neutrality, followed by drying for subsequent use.

The bacterial pellet collected by centrifugation was resuspended in 50 mL of LB liquid medium, and the pretreated carbon cloth electrode was then immersed in the suspension. The culture was incubated in a constant-temperature shaking incubator at 37 °C and 180 rpm for 24 h. Bacterial colonization on the surface of the carbon cloth fibers was promoted through this in situ growth process, thereby enabling the construction of a stable biofilm electrode.

### 2.5. Electrochemical Performance Measurements

Electrochemical measurements were performed using a CHI 760E electrochemical workstation (Chenhua Instrument Co., Ltd., Shanghai, China). All experiments were conducted in a five-neck electrochemical cell equipped with a standard three-electrode configuration: a carbon cloth (CC) electrode loaded with the catalyst (1 × 1 cm^2^) served as the working electrode, a platinum (Pt) plate as the counter electrode, and a saturated silver/silver chloride (Ag/AgCl) electrode as the reference electrode. All measurements were carried out in 80 mL of M9 buffer solution (pH 7.0) at 37 °C. The electrolyte composition consisted of 22 mM KH_2_PO_4_, 42 mM Na_2_HPO_4_, 85.5 mM NaCl, 1.0 mM MgSO_4_, and 0.1 mM CaCl_2_.

Prior to cyclic voltammetry (CV) measurements, the electrolyte was saturated with N_2_ or O_2_ for 30 min, and the scan rate was set to 50 mV/s. Linear sweep voltammetry (LSV) curves were recorded under O_2_-saturated conditions at a scan rate of 5 mV/s. Additionally, electrochemical impedance spectroscopy (EIS) was performed at an applied potential of −0.3 V (vs. Ag/AgCl).

### 2.6. Assembly and Performance Testing of MFC Full Cells

The experiments were conducted using an H-shaped dual-chamber microbial fuel cell (MFC) reactor with an effective working volume of 50 mL per chamber. The two chambers were separated by a proton exchange membrane (PEM). Carbon cloth pieces with an area of 4 cm^2^ were employed as catalyst supports for both the cathode and the anode. The anodic compartment was filled with M9 medium containing 1 M glucose and purged with high-purity N_2_ for 30 min to maintain an anaerobic environment. The catalyst used was commercial 40% Pt/C with a loading of 2 mg cm^−2^. The cathodic compartment contained a bioelectrode loaded with engineered *E. coli*, and the electrolyte was M9 medium supplemented with 4 g L^−1^ glucose. Prior to testing, the catholyte was saturated with O_2_ by purging for 30 min.

An electrochemical workstation was used to monitor the open-circuit voltage of the MFC. The variable resistance method was applied over a resistance range from 10 MΩ to 1 Ω to measure the steady-state output voltage under different load conditions. The polarization and power density curves of the MFC were plotted with current density as the abscissa and with voltage or power density as the ordinate, respectively. In addition, a Keithley digital multimeter (Keithley Instruments, Inc., Solon, OH, USA) was employed to record the output voltage stability of the system during long-term operation in real time.

### 2.7. Adenosine Triphosphate (ATP) Content Measurement

Adenosine triphosphate (ATP), serving as the “energy currency” of living organisms, is an important indicator for evaluating microbial metabolic activity. In this study, an ATP content assay kit was employed to measure and analyze the energy metabolism levels of various engineered *E. coli* strains. The detection principle is primarily based on an enzyme-catalyzed reaction mediated by creatine kinase (CK). CK catalyzes the reaction between ATP and creatine to generate phosphocreatine. Subsequently, the amount of phosphocreatine produced was determined using a phosphomolybdic acid colorimetric method at a wavelength of 700 nm. By measuring the changes in absorbance, the intracellular ATP content was calculated. This method was primarily used to assess the energy supply status of different strains.

### 2.8. Biofilm Protein Content Determination

Biofilm is a complex multiphase system formed by microbial cells and their secreted extracellular polymeric substances (EPSs), with proteins serving as a primary component. In this study, a BCA protein assay kit was employed to quantify the protein concentration of the *E. coli* biofilm formed on the carbon cloth surface. The detection principle is as follows: under alkaline conditions, proteins reduce Cu^2+^ to Cu^+^, which then specifically chelates with BCA reagent to produce a purple–blue complex. The absorbance of the complex was measured at 562 nm using a UV–visible spectrophotometer (Shimadzu (China) Co., Ltd., Shanghai, China). The total protein concentration in the samples was calculated based on a standard protein curve, allowing for the assessment of biofilm biomass and development.

## 3. Results and Discussion

### 3.1. Synthesis and Characterization of CDs

**Figure 2:**
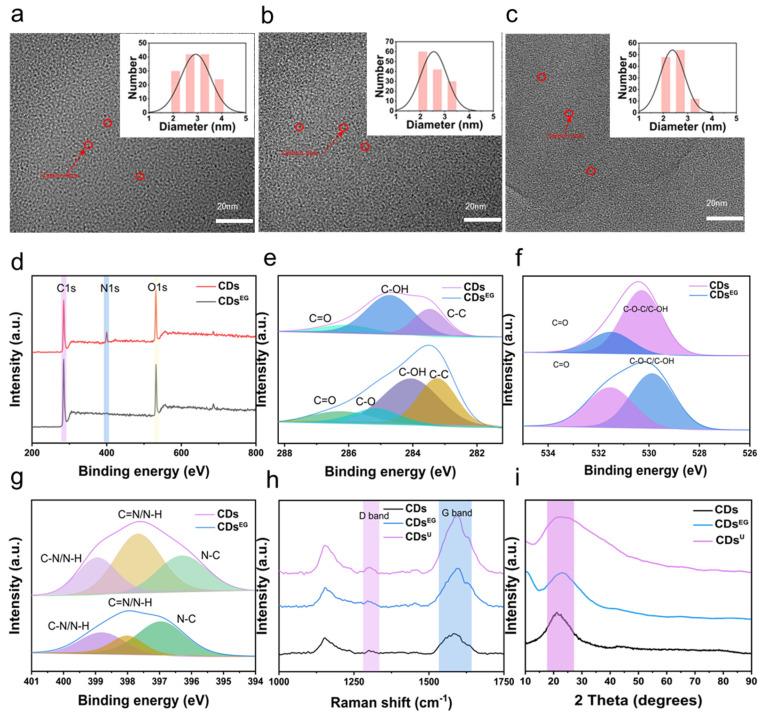
Characterization of CDs. (**a**–**c**) TEM images and particle size statistics of CDs, CDs^EG^, and CDs^U^. (**d**) XPS spectra of CDs and CDs^EG^. (**e**) C1s analysis of CDs and CDs^EG^, (**f**) O1s analysis of CDs and CDs^EG^, and (**g**) N1s analysis of CDs and CDs^EG^. (**h**) Raman spectra of CDs, CDs^EG^, and CDs^U^. (**i**) XRD analysis of CDs, CDs^EG^, and CDs^U^.

The specific synthesis process of CDs is shown in Figure S1. The microstructure and size distribution of the three samples were characterized using transmission electron microscopy (TEM) and particle size distribution histograms, and the results are shown in Figure 2a–c. The results reveal that CDs, CDs^EG^, and CDs^U^ all exhibited a quasi-spherical morphology, with average diameters of approximately ~2.7 nm, ~2.3 nm, and ~2.2 nm, respectively [22].

X-ray photoelectron spectroscopy (XPS) was employed to characterize the surface chemical composition and bonding states of the samples. Figure 2d presents the XPS survey scan spectra of CDs and CDs^EG^. In the survey spectra, the three main characteristic peaks located at 284.4 eV, 400.0 eV, and 532.0 eV correspond to the C 1s, N 1s, and O 1s orbitals, respectively, confirming the presence of carbon, nitrogen, and oxygen elements in all samples. Semi-quantitative analysis of the survey spectra indicates the surface elemental composition of CDs is C 70.2%, O 22.4%, and N 7.4%, whereas for CDs^EG^, it shifts to C 80.4%, O 18.9%, and N 0.7%. The substantial reduction in nitrogen content in CDs^EG^ is expected to have a significant influence on its catalytic performance [23].

To further investigate the chemical states of each element, high-resolution XPS spectra for C 1s, O 1s, and N 1s were subjected to peak-fitting deconvolution; the results are presented in Figure 2e–g, respectively. In the C 1s spectrum (Figure 2e), the three fitted peaks at binding energies of 284 eV, 286 eV, and 287 eV can be assigned to the C=C/C-C (sp^2^ carbon), C-OH (hydroxyl/epoxy groups), and C=O (carbonyl) functional groups, respectively. In the O 1s spectrum (Figure 2f), the two components located at 531 eV and 532 eV correspond to oxygen-containing groups, such as C=O and C-O-C/C-OH, respectively. Finally, deconvolution of the N 1s spectrum (Figure 2g) reveals three peaks in the vicinity of 399 eV, 400 eV, and 401 eV, which can be attributed to different nitrogen doping configurations, namely pyridinic nitrogen (N-C), pyrrolic nitrogen or chemisorbed nitrogen (C=N/N-H), and graphitic nitrogen (C-N/N-H), respectively [24].

The FT-IR transmittance spectrum of the synthesized carbon quantum dots (CDs) is shown in Figure S2. A broad absorption band at ~3383 cm^−1^ is attributed to the stretching vibrations of hydroxyl (-OH) or amine (-NH) groups, while the sharp peak at ~2927 cm^−1^ arises from sp^3^ C-H stretching vibrations. The high-intensity sharp feature observed at ~2019 cm^−1^ indicates stretching vibrations from shifted multi-bonded carbon systems (e.g., cumulenes or isonitriles), possibly resulting from reaction intermediates. A prominent peak at ~1664 cm^−1^ confirms the existence of highly polar carbonyl bonds (C=O) from carboxyl or amide groups. The band at ~1366 cm^−1^ likely corresponds to C-N stretching or C-H in-plane bending. Furthermore, the broad and intense band around ~1050 cm^−1^ is ascribed to C-O single bond stretching in ether or hydroxyl groups. Collectively, these results demonstrate a surface rich in hydrophilic groups (especially -COOH, -OH, and C-O-C), which accounts for the excellent water solubility of the CDs in aqueous environments.

Furthermore, Raman spectroscopy was employed to characterize the microstructure of the samples, and the results are shown in Figure 2h. All samples exhibit two typical characteristic peaks at approximately 1350 cm^−1^ and 1580 cm^−1^, corresponding to the defect-induced D-band and the graphitic G-band of carbon materials, respectively. These results indicate that the synthesized carbon quantum dots consist of both ordered graphitic domains (G-band) and structural defects/disordered regions (D-band). The integrated intensity ratio of the D-band to the G-band (ID/IG) is commonly used to evaluate the defect density or graphitic order in carbon materials. For all samples, the calculated ID/IG ratios are less than 1, indicating that ordered graphitic regions predominate. Notably, the ID/IG ratio of CDs is higher than that of CDs^EG^, suggesting that the CDs contain a greater abundance of structural defects. This observation aligns with XPS analysis, which showed higher nitrogen doping in CDs (N 7.4%). The introduction of nitrogen atoms tends to disrupt the integrity of the carbon lattice, thereby inducing more defect sites and potentially increasing the density of surface-active functional groups [25]. The analysis of the XRD patterns indicates that all three samples are typical carbon quantum dot structures, mainly composed of amorphous or turbostratic graphite (Figure 2i). The (002) peak of CDs^U^ slightly shifts to the right (towards a higher angle), indicating that its d002 interlayer spacing is slightly smaller and its degree of graphitization is slightly higher than that of CDs and CDs^EG^. CDs have the largest interlayer spacing, suggesting that their structure may be more disordered, which is consistent with the higher N content observed in the previous XPS analysis.

To further verify the absence of unreacted riboflavin precursor residues in the synthesized CDs, High-Performance Liquid Chromatography (HPLC) analysis was performed. As illustrated in Figure S4, under a detection wavelength of 267 nm, the riboflavin standard exhibited a sharp and distinct characteristic absorption peak at a retention time of approximately 9.40 min (highlighted region). However, when the CD sample was analyzed under identical chromatographic conditions, the baseline remained completely flat within this specific retention time window, with no observable characteristic peaks corresponding to riboflavin. Although the CD sample displayed several other peaks at different retention times (e.g., around 4.97 and 10.24 min), which are attributed to the intrinsic structure of the carbon dots or other reaction byproducts, the complete absence of the riboflavin peak strongly demonstrates that the synthesized CDs did not contain any significant free riboflavin residues. This chromatographic result not only confirms the purity of the sample but also effectively rules out the possibility that unreacted riboflavin molecules directly contribute to the electrochemical performance of the system.

### 3.2. Electrochemical Performance

**Figure 3:**
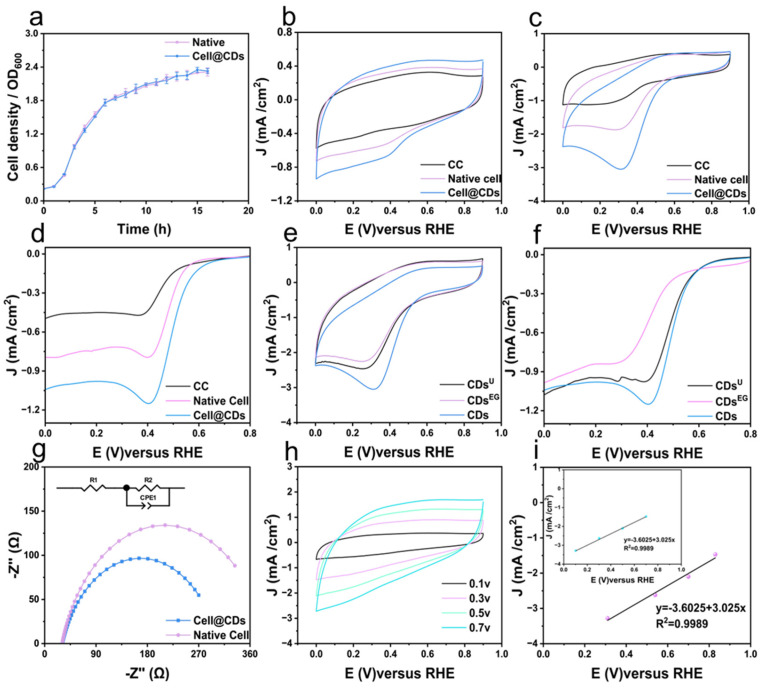
Analysis of the electrochemical performance of different *E. coli* cells. (**a**) Growth curve; (**b**) CV curves in N_2_-saturated M9 medium; (**c**) CV curves in O_2_-saturated M9 medium; (**d**) LSV curves; (**e**) CV curves of *E. coli* cells with different carbon quantum dots in O_2_-saturated M9 medium; (**f**) LSV curves of *E. coli* cells with different carbon quantum dots; (**g**) EIS Nyquist plots of different *E. coli* strains; (**h**) multi-scan rate CV curves of Cell@CDs; and (**i**) linear relationship between cathodic peak current and scan rate (the inset shows the linear relationship between the cathodic peak current and the square root of the scan rate).

The initial growth curve assays demonstrated that the introduction of CDs did not significantly affect the growth profile of *E. coli* (Figure 3a). Electrochemical evaluations revealed that the resulting *E. coli* Cell@CD composite exhibited outstanding catalytic performance for ORR within the MFC cathode. Cyclic voltammetry (CV) measurements conducted in an N_2_-saturated environment showed clear direct electron transfer (DET) electrochemical redox behavior in *E. coli* cells, which was particularly pronounced in the *E. coli* Cell@CD composite. To further assess the electrochemical performance, a standard three-electrode system was employed. Significant reduction peaks were observed, indicating that oxygen could be effectively reduced by both wild-type *E. coli* and the *E. coli* Cell@CD composite (Figure 3b). Subsequent CV tests in an O_2_-saturated environment showed that the reduction current of the *E. coli* Cell@CD composite reached 3.1 mA cm^−2^, which is much higher than that of wild-type *E. coli* at 1.9 mA cm^−2^ (Figure 3c).

Linear sweep voltammetry (LSV) measurements further demonstrated the enhanced electrocatalytic activity of the *E. coli* Cell@CD composite, which exhibited a larger diffusion-limited current density of approximately 1.1 mA·cm^−2^ compared to 0.8 mA·cm^−2^ of the native cells (Figure 3d). Comparative analysis of cells incorporated with CDs synthesized via different methods clearly indicated that the biocatalyst fabricated by riboflavin-derived CDs (Cell@CDs) outperformed those prepared through other routes (Cell@CDs^U^ and Cell@CDs^EG^) (Figure 3e,f).

To deeply analyze the charge transfer and mass transport kinetic characteristics at the biocathode interface, an equivalent circuit model of R1 + (CPE1 // (R2 + W1)) was employed to fit the Nyquist plots of catalysts prepared from different cells, as shown in Figure 3g. In this series–parallel hybrid model, the total internal resistance is synergistically determined by three core components. Specifically, the high-frequency intercept R1 represents the ohmic resistance of the system, which primarily comprises the intrinsic resistance of the conductive substrate, the ionic resistance of the electrolyte solution, and the contact resistance at the electrode–electrolyte interface. The capacitive loop in the high-to-medium frequency region is formed by the parallel combination of the constant phase element CPE1 and the impedance of the lower branch. Due to the significant surface inhomogeneity of the bioreaction interface interwoven by *E. coli* cells, carbon quantum dots, the introduction of CPE1 can accurately simulate this non-ideal double-layer capacitance dispersion effect. R2 at the bottom of the parallel branch represents the charge transfer resistance, which directly reflects the activation energy barrier for electrons transferring across membranes among the conductive skeleton, carbon quantum dots, and microbial cells to drive the oxygen reduction reaction (ORR). The Warburg impedance W1 in series with R2 quantitatively characterizes the mass transport process in the low-frequency region, corresponding to the diffusion resistance of oxygen and substrate molecules traversing through the thick biofilm and the hierarchical porous structure of the substrate. This equivalent circuit model precisely resolves the impedance distribution of each component at the biohybrid interface, providing key electrochemical kinetic evidence to substantiate that the carbon quantum dot modification accelerates transmembrane electron transfer.

Furthermore, CV tests conducted on the *E. coli* Cell@CD composite at different scan rates revealed a linear relationship between the peak current and the scan rate. Linear regression analysis yielded a high correlation coefficient (R^2^) of 0.9989. In contrast, the linear regression for the relationship between the peak current and the square root of the scan rate yielded a lower R^2^ of 0.9858. These comparative results collectively demonstrate that the oxygen reduction reaction (ORR) process is predominantly governed by surface-controlled kinetics (Figure 3h,i).

**Figure 4:**
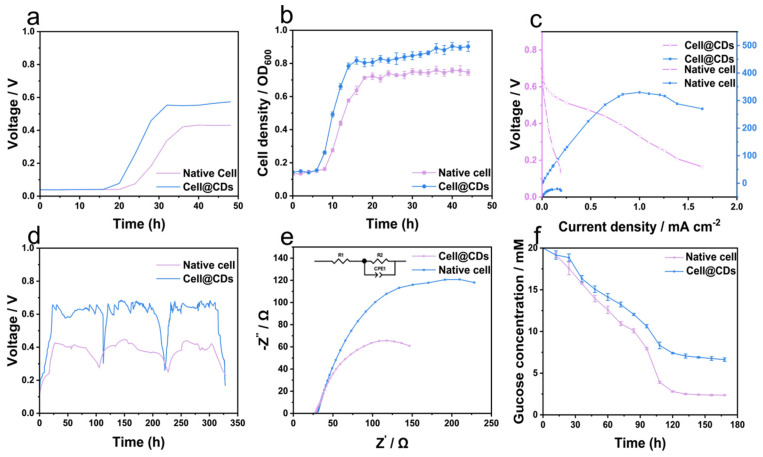
MFCs assembled using carbon cloth biocathodes loaded with different *E. coli* cells: (**a**) startup voltage curve; (**b**) whole-cell growth curve; (**c**) polarization and power density curves; (**d**) whole-cell long-term cycling stability curve; (**e**) EIS Nyquist plots of different *E. coli* strains; (**f**) glucose degradation tests in the cathode chamber using different *E. coli* strains.

To simulate the practical application of MFCs in wastewater treatment and electricity generation, a complete MFC system using glucose as the bacterial carbon source was constructed. Wild-type *E. coli* and the *E. coli* Cell@CD composite were employed as the cathode catalysts for a series of comparative tests. Analysis of the relationship between the bacterial growth curve and the output voltage in the full MFC system revealed a strong correlation: the increase in voltage corresponded with bacterial growth and stabilized as the culture entered the stationary phase. This observation indicates that the output voltage is largely determined by the microbial population density and metabolic activity (Figure 4a,b) [21,26,27].

The polarization and power density curves further confirmed the superior performance of the Cell@CD cathode. The *E. coli* Cell@CDs achieved a higher open-circuit voltage of 0.74 V, compared to 0.61 V for the wild-type *E. coli*. Notably, the maximum power density of the Cell@CD MFC reached 325 μW·cm^−2^, representing a substantial improvement over the 24 μW·cm^−2^ achieved by the wild-type MFC (Figure 4c). Long-term stability tests conducted with a fixed external load resistor demonstrated that the Cell@CD MFC maintained a consistently higher average output voltage of 0.61 V (Figure 4d) [28,29].

To further investigate the extracellular electron transfer (EET) efficiency, electrochemical impedance spectroscopy (EIS) was performed on MFCs equipped with the different biocathodes. The EIS results indicate that the Cell@CD MFC exhibited both a lower charge transfer resistance and a reduced solution resistance compared to the native *E. coli* MFC. Furthermore, equivalent circuit simulations were conducted to fit the impedance data, and the corresponding parameters are summarized in Table S4. These findings collaboratively suggest significantly accelerated electron transfer kinetics, both among bacterial cells and between the bacteria and the electrode, within the Cell@CD system (Figure 4e) [30].

Consistent with the enhanced electron transfer, the glucose consumption rate in the cathode chamber of the Cell@CD MFC was significantly accelerated, attributable to enhanced electrode respiration, which likely increased metabolic flux and stimulated cell growth. Analysis of the glucose consumption kinetics confirmed that the Cell@CD MFC depleted glucose more rapidly (18 mM within 120 h) than the native cell MFC (14 mM), highlighting its more vigorous metabolic activity (Figure 4f) [31].

### 3.3. Catalytic Mechanism

**Figure 5:**
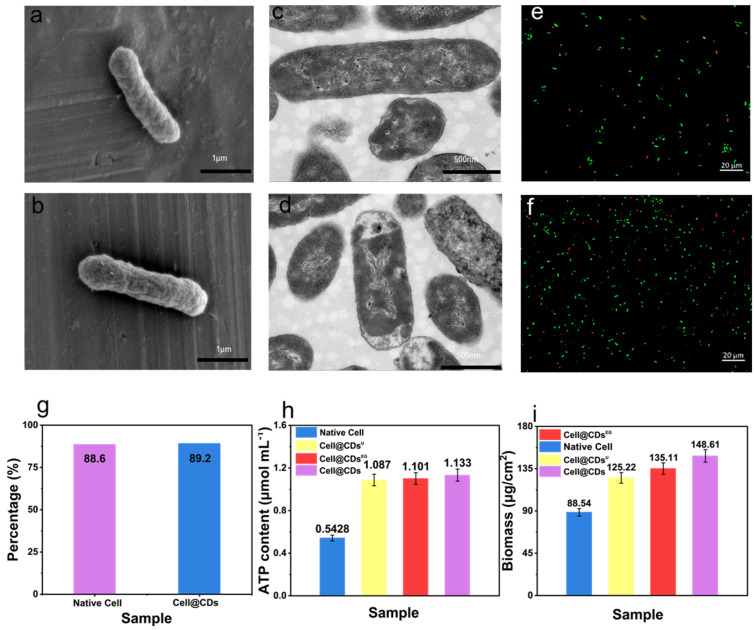
Analysis of biological characteristics of different *E. coli* cells: (**a**) SEM image of native cells; (**b**) SEM image of Cell@CDs; (**c**) TEM image of native cells; (**d**) TEM image of Cell@CDs; (**e**) CLSM image of native cells; (**f**) CLSM image of Cell@CDs; (**g**) viability statistics based on CLSM; (**h**) ATP levels of different *E. coli* strains; (**i**) protein content determined by BCA assay for different *E. coli* strains.

Scanning electron microscopy (SEM) was performed on wild-type *E. coli* and carbon dot-incorporated *E. coli* (Cell@CDs) to verify that the incorporation of carbon quantum dots did not compromise the bacteria’s structure. Compared to the native cells (Figure 5a), Cell@CDs (Figure 5b) revealed that both maintained the typical oval morphology of *E. coli*, with no observable structural changes, consistent with the expected outcome [18,32].

To verify the successful internalization of the CDs, transmission electron microscopy (TEM) analysis was conducted. Compared to the native cells (Figure 5c), the TEM images of the Cell@CD composite (Figure 5d) clearly showed numerous dark aggregates inside the bacterial cells, providing direct evidence of successful penetration of CDs into the cells [18].

To intuitively compare the bacterial abundance within the constructed biofilms, 3D laser scanning confocal microscopy (CLSM) imaging was performed on both samples (Figure S4). Compared to the control biofilm formed by pure bacteria on the carbon cloth (Figure S4a), the biofilm integrated with carbon dots (CDs) (Figure S4b) exhibited a significantly enhanced and more uniform green fluorescence signal. Crucially, this discrepancy in fluorescence intensity does not originate from the CDs themselves; rather, it accurately reflects a substantial increase in bacterial population within the biofilm. Under identical cultivation and rinsing protocols, the incorporation of CDs appears to provide a more biocompatible interface that facilitates bacterial attachment, thereby culminating in a denser and more robust biofilm architecture. The elevated green fluorescence density firmly demonstrates that CDs effectively promote bacterial colonization and proliferation on the carbon cloth substrate, establishing a more potent bioelectrochemical catalytic foundation in terms of both microstructure and biomass quantity.

Both the native and Cell@CDs groups exhibited strong green fluorescence (indicative of live cells) and weak red fluorescence (indicative of dead cells), demonstrating that the incorporation of CDs had negligible cytotoxic effects on *E. coli*. Quantitatively, the density of Cell@CDs cells adhered to the carbon cloth was significantly higher than that of the native cells, indicating that the CDs enhanced bacterial adhesion and promoted biofilm formation on the electrode surface (Figure 5e,f). Subsequent analysis using Imaris software (Imaris 10.1) revealed high and comparable cell viability rates of 88.6% for the wild-type (WT) and 89.2% for the Cell@CD composite, further confirming the excellent biocompatibility of the CDs (Figure 5g) [33].

To evaluate the energy metabolism level of *E. coli*, ATP content was assessed, as ATP serves as the universal energy currency and higher ATP levels indicate increased cellular metabolic activity. Quantitative measurement using an ATP assay kit (Mlbio, ml092826, Shanghai Enzyme-linked Biotechnology Co., Ltd., Shanghai, China) showed that the ATP level in Cell@CDs (1.133 μmol·mL^−1^) was more than twice that of native cells (0.5428 μmol·mL^−1^). Furthermore, ATP level in Cell@CDs was slightly higher than those in the control samples, Cell@CDs^U^, and Cell@CDs^EG^ (Figure 5h) [34,35].

Membrane protein content was also measured using the BCA method. The results show that Cell@CDs contained 148.61 μg·cm^−2^, approximately 1.68 times higher than that of native cells (88.54 μg·cm^−2^). Moreover, the protein content in Cell@CDs was slightly higher than that in the control samples. These results demonstrate that (1) the experimental sample had a significantly greater biomass than the native sample within the same unit area of carbon cloth and (2) membrane proteins, which serve as electron carriers, contribute to enhanced electron transfer capability (Figure 5i) [36].

This study developed an effective strategy to enhance bioelectricity generation and pollutant degradation by synthesizing CDs through a simple and eco-friendly method, integrating them with safe *E. coli* and applying the resulting system in microbial fuel cells. The system exhibited performance among the upper tier of current research in this field, as supported by comparative performance data presented in Tables S1 and S2 [37,38,39,40,41,42,43].

### 3.4. Engineering Implications and Practical Relevance

In resource-intensive industries, this system can be directly implemented for organic wastewater treatment. Leveraging the superior metabolic capabilities of engineered *Escherichia coli*, the system enables the direct conversion of chemical energy into electrical power through an enhanced oxygen reduction reaction (ORR) while simultaneously degrading complex organic pollutants, such as sugars and organic acids, present in industrial effluents. This dual-effect mechanism of “simultaneous power generation and water purification” provides a viable technological pathway for the development of low-energy or even energy-negative industrial wastewater treatment plants. Currently, industrial fuel cells rely heavily on noble metal catalysts like platinum (Pt), which are not only prohibitively expensive but also highly susceptible to poisoning by impurities such as sulfides. Commercial Pt/C catalysts currently cost over $30,000 per kilogram, accounting for nearly 40% of the total microbial fuel cell (MFC) stack cost. In stark contrast, the industrial cost of riboflavin is approximately $30 per kilogram. By utilizing inexpensive and readily available riboflavin as a precursor for carbon quantum dot synthesis, combined with the large-scale fermentation potential of *E. coli*, this study offers a low-cost, sustainable, and environmentally friendly biocatalyst solution. This advancement is of significant importance for lowering the economic barriers to the large-scale industrial deployment of microbial fuel cells.

Despite the promising performance of the CD-modified *E. coli* biohybrid cathode, several limitations remain to be addressed in future studies. First, while *E. coli* is highly versatile for genetic engineering and biomass degradation, its intrinsic extracellular electron transfer (EET) capability is lower than that of naturally electroactive bacteria (e.g., *Shewanella* or *Geobacter*), which may cap the upper limit of the long-term current output. Second, the long-term operational stability and biocatalyst viability under continuous-flow, harsh industrial wastewater conditions require further validation, as microbial activity can be susceptible to complex environmental fluctuations.

## 4. Conclusions

In summary, this study reports a facile, environmentally friendly, and economical strategy for constructing a novel whole-cell biocatalyst (Cell@CDs) and demonstrates its exceptional MFC cathode performance. Comprehensive characterizations reveal that the incorporation of CDs orchestrates a multi-dimensional enhancement of the biohybrid system. Specifically, CDs significantly boosted the intracellular metabolic activity, as evidenced by a more than two-fold increase in ATP levels (1.133 μmol·mL^−1^ vs. 0.543 μmol·mL^−1^ in wild-type) and a 1.68-fold increase in membrane protein content (148.61 μg·cm^−2^ vs. 88.54 μg·cm^−2^ in wild-type), which are crucial for enhanced electron transfer. Electrochemically, Cell@CDs exhibit superior ORR activity with a high current density of 3.1 mA·cm^−2^ and an onset potential of 0.67 V. When integrated into MFCs, it delivers a remarkable maximum power density of 325 μW·cm^−2^, vastly outperforming wild-type *E. coli* (24 μW·cm^−2^). This superiority stems from CD-promoted biofilm formation, reduced interfacial charge transfer resistance (EIS-verified), and efficient transmembrane electron conduction networks. This work provides valuable insights for high-performance whole-cell biocatalyst design and advances the practical application of bioelectrochemical systems.

## Data Availability

The original contributions presented in this study are included in the article/Supplementary Materials. Further inquiries can be directed to the corresponding authors.

## Supplementary Materials

The following supporting information can be downloaded at https://www.mdpi.com/article/10.3390/molecules31122039/s1: Figure S1. Hydrothermal Synthesis Pathways of Carbon Dots. Figure S2. The FT-IR transmittance spectrum of the synthesized carbon quantum dots (CDs). Figure S3. Presents the High-Performance Liquid Chromatography (HPLC) chromatograms of the riboflavin standard and the synthesized carbon dots (CDs). Figure S4. 3D CLSM images of biofilms on carbon cloth: (a) Native, and (b) Cell@CDs. Figure S5. UV-vis absorbance standard calibration curve of carbon quantum dots. Table S1. Performance comparison of several MFC half-cells. Table S2. Performance comparison of several MFC full-cells. Table S3. Fitting results of different cathode electrodes in half-cells derived from Nyquist plots. Table S4. Fitting results of different cathode electrodes in full-cells derived from Nyquist plots.
