# Supplementary material for: Engineered Escherichia coli Modified with Carbon Quantum Dots as a High-Performance Cathode Catalyst for Microbial Fuel Cells
Source: Molecules. 2026 Jun 11;31(12):2039. doi: 10.3390/molecules31122039 (PMC13304498; doi:10.3390/molecules31122039)
Supplement: Supplementary file 1 [file molecules-31-02039-s001.zip › molecules-4281449-supplementary.pdf]

1. Materials and Methods

1.1. Strain Activation and Culture

The *Escherichia coli* BL21 (DE3) strain preserved at −80 °C was inoculated into 5 mL of LB liquid medium and incubated in a shaking incubator at 37 °C and 180 rpm until the logarithmic growth phase (OD600 = 0.6–0.8). Subsequently, 1 mL of the bacterial culture was transferred into 100 mL of fresh LB liquid medium at a 100-fold dilution for scale-up culture. When the bacteria reached the late logarithmic growth phase (OD_{600} = 1.5–1.6), the culture was centrifuged at 6000 rpm for 5 minutes at 4 °C. The supernatant was discarded, and the resulting bacterial pellet was collected for further use.

1.2. Bacterial Growth Curve Analysis

To determine the bacterial growth curve, the strain preserved at −80 °C was inoculated into 5 mL of LB liquid medium and incubated in a shaking incubator at 37 °C and 180 rpm until the mid-logarithmic phase (OD_{600} = 0.6–0.8). Subsequently, 1 mL of the bacterial culture was transferred into 200 mL of fresh LB medium and further cultured under the same conditions (37 °C, 180 rpm). The time of inoculation was designated as the starting point (time zero), and the initial OD_{600} value was recorded using a UV–visible spectrophotometer. Thereafter, the OD_{600} of the sample was measured every hour for a continuous period of 16 hours. Finally, the bacterial growth curve was plotted with culture time as the abscissa and OD_{600} as the ordinate to evaluate the growth kinetics of the bacteria.

1.3. Synthesis of Carbon Quantum Dot-Engineered Escherichia coli

To restore the viability of the preserved *Escherichia coli* BL21(DE3) strain, it was first inoculated into 5 mL of LB liquid medium and cultured overnight (at least 12 hours) in a constant-temperature shaking incubator at 37 °C. Subsequently, 200 μL of this activated bacterial culture was transferred into a conical flask containing 200 mL of fresh LB medium and further cultured until the optical density at 600 nm (OD_{600}) reached approximately 1.5. At this point, the bacterial cells were harvested by centrifugation (5000 rpm, 5 min) and stored for subsequent experiments.

The harvested E. coli cells were washed twice with ultrapure water. Subsequently, 100 μL of the carbon quantum dot solution (27 mg/mL) was added into 10 mL of ultrapure water, and the previously centrifuged bacterial pellet was thoroughly resuspended in this mixture. The mixed bacterial suspension was incubated in a constant-temperature shaking incubator for 2 hours to allow sufficient interaction and incorporation of the carbon quantum dots into the bacteria. The same incorporation procedure was applied for all three types of carbon quantum dots. The resulting *E. coli* cells incorporated with the three different carbon quantum dots were designated as Cell@CDs, Cell@CDs^{EG}, and Cell@CDs^{U}, respectively.

1.4. Confocal laser scanning microscopy (CLSM)

Confocal laser scanning microscopy (CLSM) was employed to obtain high-resolution images of the biofilm on the electrode surface. This technique utilizes its excellent optical sectioning capability to acquire optical sections at different sample depths by adjusting the focal plane, thereby enabling non-destructive observation and three-dimensional reconstruction of the cellular and biofilm architecture. In this study, the samples were la*beled using a live/dead fluorescence staining technique to enable real-time observation of* bacterial morphology and physiological activity at the subcellular level.

Cell viability and morphology of both native and engineered *E. coli* strains were assessed using a live/dead cell viability kit. Briefly, 1 mL of bacterial culture at the late logarithmic growth phase was collected by centrifugation at 6000 rpm for 5 min. The resulting pellet was then resuspended in 1 mL of physiological saline (0.85% NaCl). A pre-prepared staining working solution, consisting of 1 μL NucGreen and 2 μL EthD-III premixed in 8 μL of physiological saline, was added to the bacterial suspension. The mixture was incubated for 15 min at room temperature in the dark. In this staining system, NucGreen (green fluorescence) labels all bacterial cells, while EthD-III (red fluorescence) specifically labels membrane-compromised (dead or damaged) cells, thereby enabling both qualitative and quantitative analysis of cell viability.

1.5. High-Performance Liquid Chromatography (HPLC)

The quantitative analysis of riboflavin and carbon dots (CDs) was performed on a Waters 2695 High-Performance Liquid Chromatography (HPLC) system (Waters, USA) equipped with a Waters 2998 Photodiode Array (PDA) Detector. Chromatographic sepration was achieved using an XBridge C18 column (4.6 mm x 250 mm, 5 μm particle size; Waters, USA) maintained at a constant temperature of 30 degrees Celsius. The mobile phase consisted of a mixture of methanol and 20 mM disodium hydrogen phosphate (Na_2HPO_4) solution (65:35, v/v) under isocratic elution conditions at a flow rate of 1.0 mL/min. The injection volume was set to 10 μL, and the PDA detector was configured to scan a wavelength range of 190–800 nm, with quantitative monitoring performed at the specific absorption wavelength of 267 nm.

For sample preparation, a standard stock solution of riboflavin (0.22 mg/mL) was prepared by dissolving riboflavin powder in 0.1 M hydrochloric acid (HCl) solution. Prior to HPLC injection, the stock solution was diluted 100-fold using the mobile phase to a final concentration of 2.2 μg/mL. Similarly, the carbon dots solution (0.27 mg/mL) was diluted 100-fold with the mobile phase to a final concentration of 2.7 μg/mL. All standard solutions and samples were filtered through a 0.22 μm syringe filter before analysis to remove potential particulates.

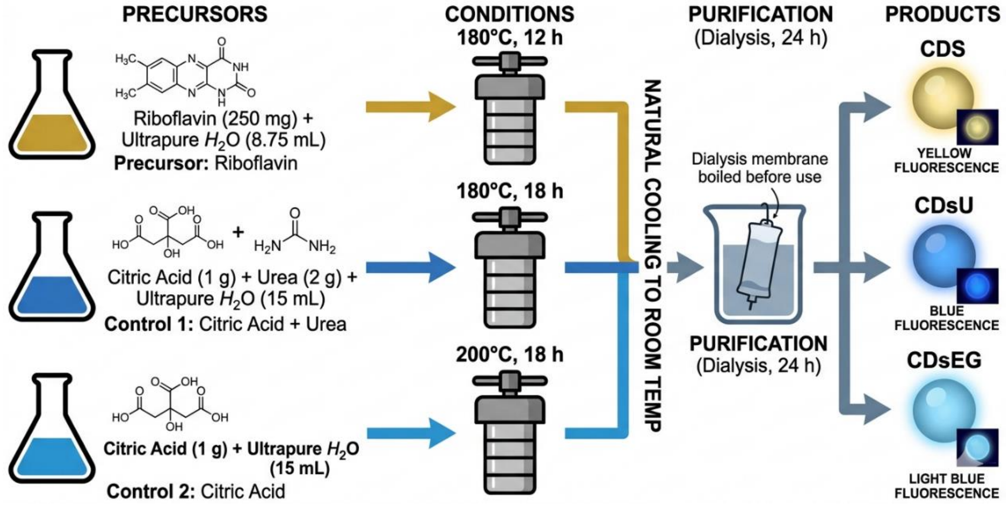

Figure S1. Hydrothermal Synthesis Pathways of Carbon Dots.

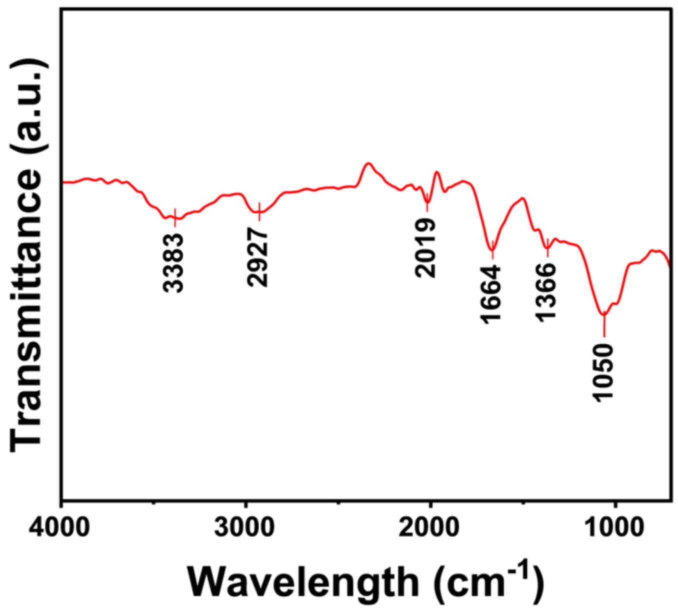

Figure S2. The FT-IR transmittance spectrum of the synthesized carbon quantum dots (CDs).

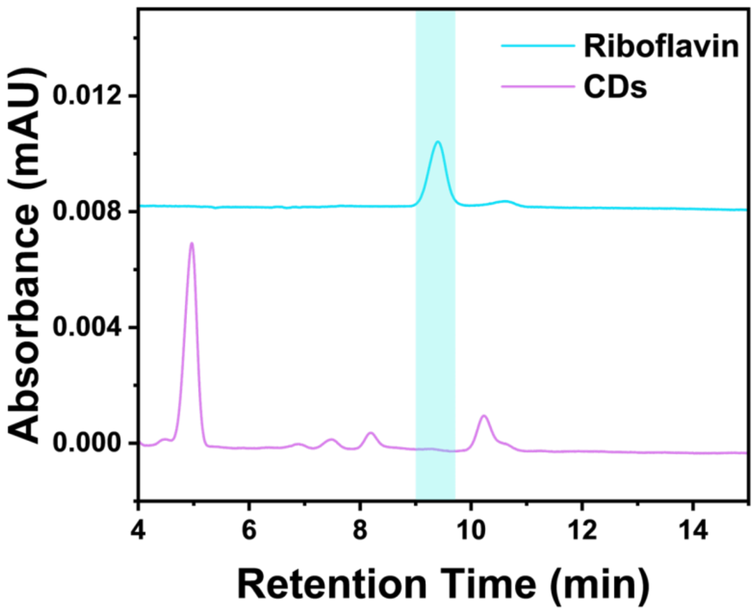

Figure S3. Presents the High-Performance Liquid Chromatography (HPLC) chromatograms of the riboflavin standard and the synthesized carbon dots (CDs).

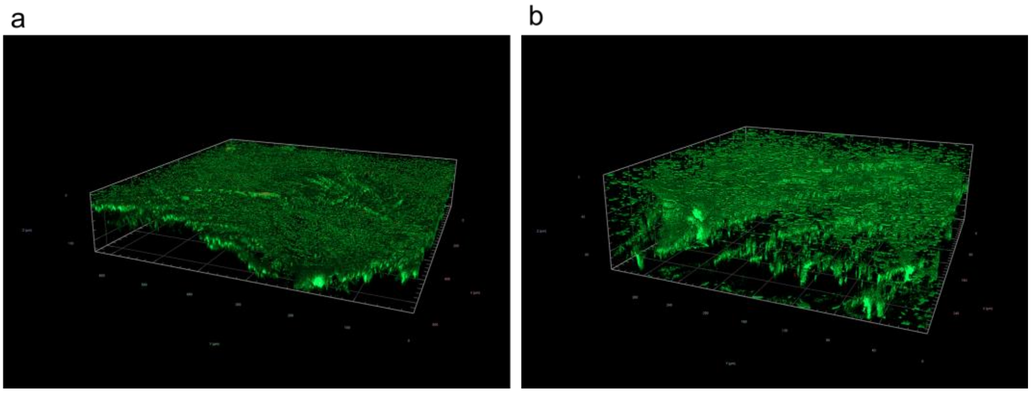

Figure S4. 3D CLSM images of biofilms on carbon cloth: (a) Native, and (b) Cell@CDs.

The initial CQD solution (27 mg/mL) was first diluted 200-fold to ensure that the absorbance fell within the linear measurable range of the UV-vis spectrophotometer. Subsequently, the UV-vis absorption spectrum of the CQDs was acquired using UV-vis spectrophotometry, and a standard calibration curve was established using a gradient of CQD concentrations. To determine the uptake amount, we measured the absorbance of the CQD solution both prior to feeding the *E. coli* and of the resulting supernatant after the incubation (feeding) process. The corresponding CQD concentrations were deduced from the calibration curve to calculate the final cellular uptake. The characteristic absorption peak of the CQDs was identified at 357 nm. Based on the gradient concentrations, the standard calibration curve for CQD absorbance was constructed, as shown in Figure S5. Five parallel replicates were performed for each measurement. The average optical density at 357 nm (OD_{357}) before bacterial uptake was determined to be 3.015, corresponding to a diluted concentration of 0.1357 mg/mL and an initial concentration of 27.14 mg/mL (before the 200-fold dilution). After bacterial incubation, the average OD_{357} of the supernatant dropped to 2.9237, yielding a diluted concentration of 0.1317 mg/mL and a pre-dilution concentration of 26.34 mg/mL. Given that the total loading volume of the CQD solution was 100 μL, the total uptake amount of CQDs by the *E. coli* on the electrode was calculated to be 0.08 mg.

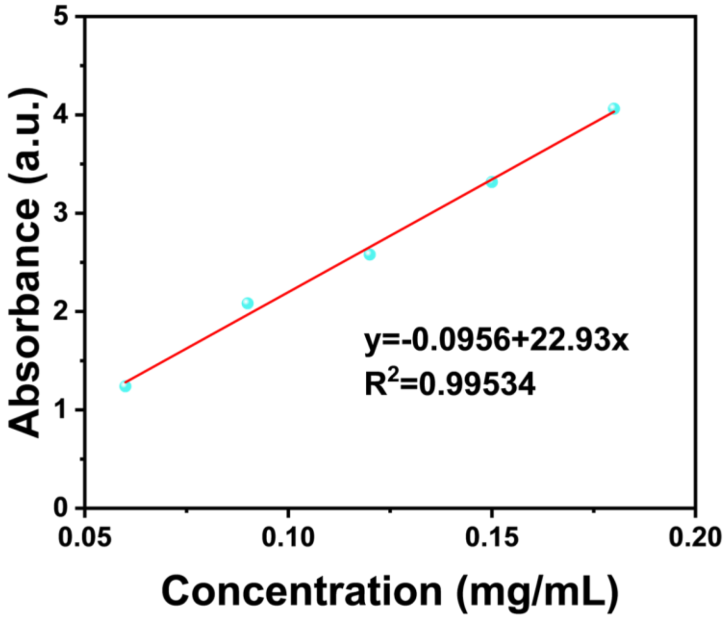

Figure S5. UV-vis absorbance standard calibration curve of carbon quantum dots.

Table S1. Performance comparison of several MFC half-cells

**Catalyst**	**Current density ($mA\ cm^{-2}$)**	**Peak potential (V)**	**References**
Laccase	0.6 (1 mV • s^{-1})	0.54 (vs. Ag/AgCl)	[37]
BOD	0.025 (5 mV • s^{-1})	0.55 (vs. Ag/AgCl)	[28]
Functionalized red blood cells	1.96 (10 mV • s^{-1})	0.53 (vs. RHE)	[26]
Au@Pt	0.17 (10 mV • s^{-1})	0.83 (vs. RHE)	[39]
Pt/C	0.16 (10 mV • s^{-1})	0.75 (vs. RHE)	[39]
Compost leachate	0.15 (1 mV/s^{-1})	−0.2 (vs. SCE)	[40]
**Cell@CDs**	**3.1 (50mV/s^{-1})**	**0.63 (vs. RHE)**	**This work**

Table S2. Performance comparison of several MFC full-cells

**Anode catalyst**	**Cathode catalyst**	**Power density ($\mu W\ cm^{-2}$)**	**References**
S. oneidensis MR-1@SP	$K_3[Fe(CN)_6]$	321	[7]
S. oneidensis MR-1@Ag	Laccase	36.1	[41]
Pt/C	Engineered Bacillus natto@sPDA	412	[42]
Glucose oxidase	Hyaluronate-Au@Pt	15.8	[39]
Activated sludge	Fe-NC@CBC	64.1	[43]
**Pt/C**	**Cell@CDs**	**325**	**This work**

The physical significance of each element in the equivalent circuit model is defined as follows: R1 represents the sum of the intrinsic electrode, electrolyte, and interfacial contact resistances; R2 denotes the diffusion resistance of reactants within the biofilm as well as the charge transfer resistance between the biofilm and the electrode; and Rt refers to the total internal resistance of the system.

Table S3. Fitting results of different cathode electrodes in half-cells derived from Nyquist plots.

Cathods	R1($\Omega\cdot cm^{-2}$)	Error (%)	R2($\Omega\cdot cm^{-2}$)	Error (%)
Cell@CDs	30.83	0.45563	274.2	1.3254
Native	30.54	1.2324	359.9	2.1627

The physical significance of each element in the equivalent circuit model is defined as follows: R1 represents the sum of the intrinsic electrode, electrolyte, and interfacial contact resistances; R2 denotes the diffusion resistance of reactants within the biofilm as well as the charge transfer resistance between the biofilm and the electrode; and Rt refers to the total internal resistance of the system.

Table S4. Fitting results of different cathode electrodes in full-cells derived from Nyquist plots.

Cathods	R1($\Omega\cdot cm^{-2}$)	Error (%)	R2($\Omega\cdot cm^{-2}$)	Error (%)
Cell@CDs	28.05	0.3064	189.4	1.5086
Native	30.69	0.54536	313.7	1.8527
